# Perspectives on next-generation secondary wastewater treatment for potable reuse applications

**DOI:** 10.2166/wrd.2026.134

**Published:** 2026-03-04

**Authors:** Raven C. Althouse, Harmita Golwala, Michael A. Saldana, Marisol Cira, Christelle Sawaya, Shu Ying Wee, Binglin Guo, Sarah E. Philo, Adam L. Smith

**Affiliations:** Sonny Astani Department of Civil and Environmental Engineering, University of Southern California, 3620 South Vermont Avenue, Los Angeles, California 90089, United States

**Keywords:** biotechnology, compatibility, next-generation, nutrient removal, potable reuse

## Abstract

The growing implementation of indirect and direct potable reuse (IPR and DPR) highlights the need for robust wastewater treatment to strengthen the performance and reliability of downstream advanced water purification facilities (AWPFs). Next-generation secondary waste-water treatment processes are now being considered to enhance compatibility in potable reuse schemes. This perspective analyzes the role of competing next-generation biotechnologies such as integrated fixed-film activated sludge (IFAS) and the moving bed biofilm reactor (MBBR), membrane biofilm reactor (MBfR), Modified Ludzack-Ettinger membrane bioreactor (MLE-MBR) and anaerobic membrane bioreactor (AnMBR) upstream of reverse osmosis (RO)- and activated carbon (AC)-based AWP, and their ability to enhance contaminant removal and improve overall efficiency of AWP. Both MLE-MBR and AnMBR significantly enhance the removal of suspended solids, organics, and microbial contaminants through membrane filtration. AnMBR offers the possibility of net energy-positive operation but does not remove nitrogen, limiting compatibility with AWP. MLE-MBR removes nitrogen but has significantly greater energy demand due to aeration and mixed liquor return pumping. Overall, next-generation biotechnologies provide flexible and effective solutions to enhance secondary effluent quality, supporting safe and regulation-compliant IPR and DPR applications.

## Graphical Abstract

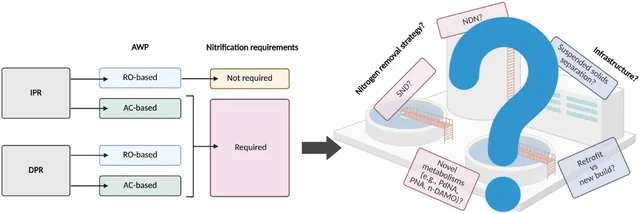

## 1. INTRODUCTION

The implementation and advancement of global water management strategies is essential for ensuring water security and promoting societal and environmental resilience. Over the past two decades, coordinated advancements in policy frameworks, technological innovation, and international cooperation have significantly expanded access to clean water. The proportion of the global population with access to safely managed drinking water increased from 61% in 2000 to 74% in 2024, while coverage of safely managed sanitation services grew from 48% in 2015 to 58% in 2024 (World Health Organization and the United Nations Children’s Fund 2025). However, a changing climate threatens to decelerate this progress by creating instability in freshwater resources across developed and developing countries alike. Global freshwater demand has increased by approximately 1% annually since the early 1980s, driven by agricultural withdrawals (~72%), followed by industrial (~15%) and domestic or municipal consumption (~13%) (United Nations Environmental Programme 2016; UNESCO World Water Assessment Programme 2024; United Nations Department of Economic and Social Affairs 2025). Meanwhile, as demands increase, satellite measurements indicate that the total global surface and subsurface freshwater volumes declined by approximately 1,200 cubic kilometers between 2002–2014 and 2015–2023 (Rodell *et al*. 2024). This convergence of rising demand and diminishing supply creates a pressing need to develop and implement water reuse systems, even in regions traditionally considered water rich.

Although seawater desalination is a widely used process globally, the United States has predominantly relied on potable water reuse due to its cost-effectiveness, technological advancements, and regulatory frameworks that support sustainable water management (U.S. Environmental Protection Agency 2017; Birch & Gall 2024; USDAnalytics 2025). Potable water reuse, or the process of using reclaimed wastewater for drinking water, offers a valuable means of water resource retention within an engineered system. However, as of 2024, only 8% of global industrial and domestic wastewater is treated with the intention of reuse (World Bank 2025), with only 48 operational facilities worldwide providing purified recycled water for potable reuse (Water Services Association of Australia 2024), making it a largely untapped global freshwater resource. Due to its relative underutilization, a lack of guidance or regulatory framework for potable reuse projects has been identified by experts as a barrier to program implementation. While water reuse regulations are predominantly driven by public health concerns of pathogen control and chemical exposure, significant complexity emerges from variations in regulatory standards and treatment processes shaped by non-health factors, including scale of implementation, environmental discharge constraints, statutory scope, and political climate (USC ReWater Center and Trussell 2024). As documented in a 2024 national survey by Trussell (Pasadena, CA, USA), these location-specific differences influence not only treatment technology selection but also the stringency of contaminant removal requirements, which may extend beyond federal Safe Drinking Water Act and Clean Water Act standards (USC ReWater Center and Trussell 2024). Therefore, water reuse necessitates individualized strategies rather than a single uniform framework, and the water reuse treatment train must be adaptable to local conditions rather than following a single standard template.

Currently, only two states in the United States, California and Colorado, have approved direct potable reuse (DPR) regulations for municipal wastewater, although Texas and Oklahoma permit case-by-case DPR projects, and 14 states have established indirect potable reuse (IPR) regulations (U.S. Environmental Protection Agency 2023; USC ReWater Center & Trussell 2024). However, these numbers are expanding rapidly as water-stressed regions increasingly recognize waste-water as an untapped reliable water source and potable reuse as a viable solution to intensifying local water insecurities (U.S. Environmental Protection Agency 2021). The growing momentum toward IPR and DPR implementation necessitates a fundamental rethinking of secondary wastewater treatment to achieve optimal contaminant removal before advanced water purification (AWP). Despite the critical importance of upstream treatment processes, secondary wastewater treatment has historically received insufficient attention in potable reuse planning, often being treated as a fixed component rather than an optimization opportunity. This perspective examines the role of competing next-generation secondary wastewater treatment biotechnologies prior to AWP, specifically highlighting their capacity to remove contaminants, providing perspective and recommendations for selecting and improving these technologies within potable reuse treatment trains.

While recent reviews have extensively cataloged emerging biotechnologies and their capabilities in wastewater applications (Sangamnere *et al*. 2023; Das *et al*. 2025; Islam 2025; Jayabal & Sivanraju 2025; Shamshad & Ur Rehman 2025), a critical knowledge gap persists in comparing these technologies within the specific context of potable reuse and their compatibility with downstream AWP processes. Existing literature evaluates biotechnologies in isolation, assessing removal efficiencies, energy conservation, and environmental impacts (Khalidi-Idrissi *et al*. 2023; Kim & Criddle 2023; Shamshad & Ur Rehman 2025), yet fails to provide an integrated decision-making framework in the context of potable reuse. This perspective offers unique insights into potable reuse treatment trains, examining the role of competing established and emerging next-generation secondary wastewater treatment biotechnologies prior to AWP, specifically highlighting their capacity to remove regulated contaminants, infrastructural constraints, energy demands, and microbial metabolic diversity, while providing perspective and recommendations for selecting and improving these technologies within the bounds of specific downstream AWP processes.

## 2. POTABLE REUSE AND ADVANCED WATER PURIFICATION

**Figure 1 |:**
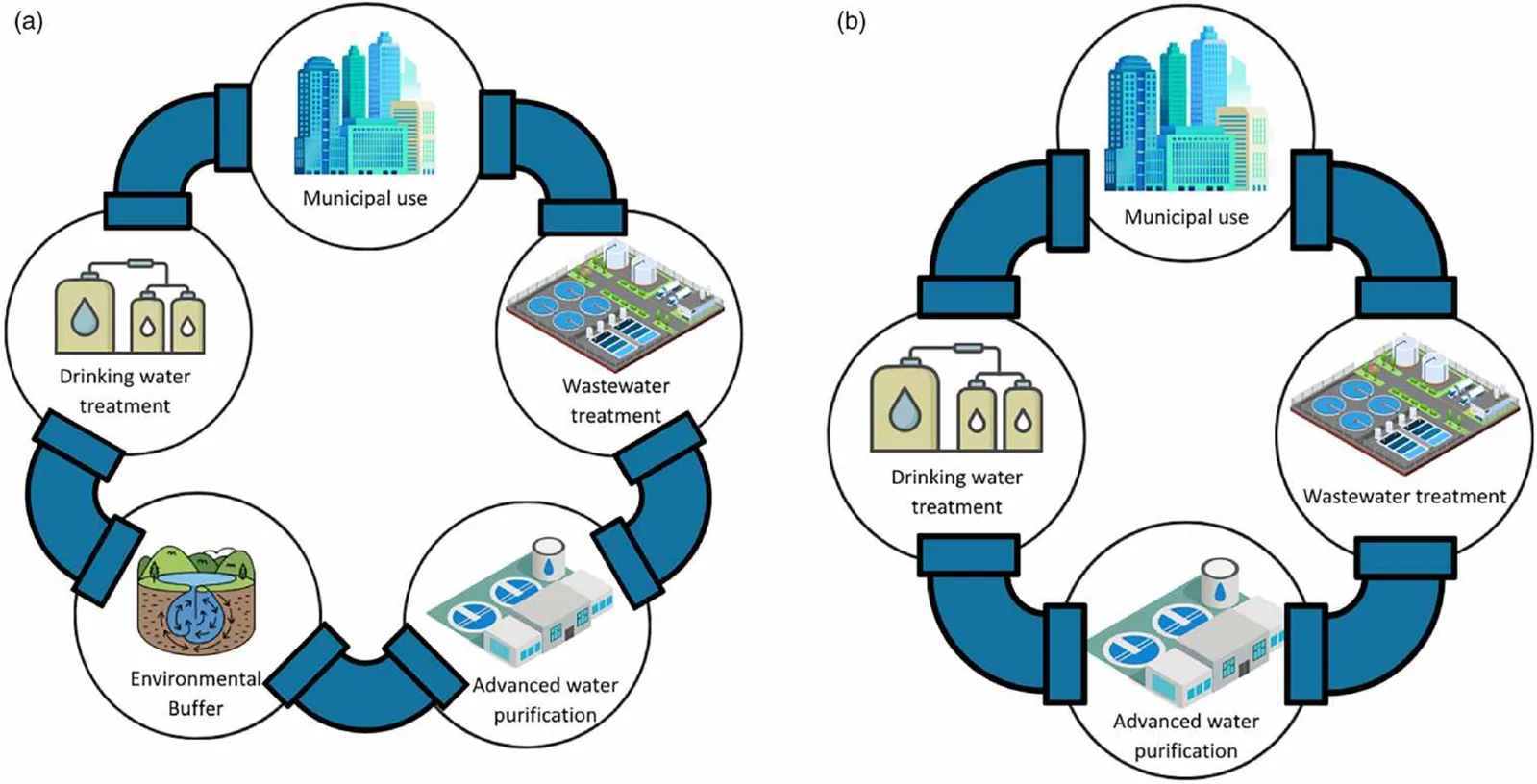
Potable reuse frameworks for (a) indirect potable reuse and (b) direct potable reuse.

Water reuse systems are multi-barrier treatment processes that prioritize resiliency, redundancy, and robustness to ensure public health and safety (Pecson *et al*. 2015). Here, ‘potable reuse’ refers to the publicly acknowledged and intentional use of reclaimed wastewater for drinking purposes, where ‘reclaimed wastewater’ has been treated through preliminary, primary, secondary, tertiary, and advanced water purification processes at a water resource recovery facility (WRRF). IPR utilizes an environmental buffer, such as a subsurface aquifer or surface reservoir, that is deliberately augmented with reclaimed wastewater providing public health protection in the form of retention time and dilution before distribution (Figure 1(a)). DPR bypasses this environmental buffer and instead introduces reclaimed wastewater directly from the advanced water purification facility (AWPF) into the water supply or drinking water distribution system (Figure 1(b)).

### 2.1. Advanced water purification for potable reuse treatment trains

AWPFs utilize integrated physical, chemical, and biological processes to further treat wastewater effluent, thereby ensuring compliance with rigorous quality standards necessary for safe reuse. These facilities employ complex multi-process systems that vary considerably between locations, with numerous configuration options available to meet site-specific effluent quality requirements (Gerrity *et al*. 2013; U.S. Environmental Protection Agency 2015, 2017). However, widely applied designs generally fall under one of two categories: (1) reverse osmosis (RO)-based treatment trains, or (2) activated carbon (AC) based trains utilizing biological activated carbon (BAC) and/or granular activated carbon (GAC). RO-based treatment trains reject dissolved solids and chemical contaminants via filtration, while AC-based trains employ biological processes and adsorption for chemical contaminant removal. Depending on regional terminology, RO- and AC-based systems may be termed ‘membrane-based’ or ‘carbon-based advanced treatment’ (CBAT), respectively; however, the former nomenclature is used here.

A leading example of an RO-based AWPF is the upgraded Groundwater Replenishment System at the Orange County Water District (OCWD) in California (USA) for IPR. This AWPF accepts treated wastewater from the Orange County Sanitation District (OC San), and provides three barriers prior to distribution to injection wells and percolation basins: (1) microfiltration (MF) targeting suspended solids, bacteria, protozoa, and some viruses; (2) RO removing dissolved chemicals, pharmaceuticals, remaining viruses, and salts; and (3) advanced oxidation processes (AOP) to disinfect and destroy any trace organics still remaining in the water (Burris 2022). Alternatively, the Sustainable Water Initiative for Tomorrow (SWIFT) Research Center (Virginia, USA) is an AC-based AWP pilot facility designed for groundwater replenishment, in which both RO- and AC-based AWP trains have been studied for the plant’s treatment objectives, resulting in permanent installation of AC-based AWPFs (Amos 2018). Secondary wastewater effluent is fed to the SWIFT AWPF where it undergoes (1) flocculation and sedimentation targeting suspended solids, (2) ozonation to break down organic material and provide disinfection, (3) biologically active filtration utilizing microbiological activity to remove dissolved organic compounds while filtering out suspended particles and pathogens, (4) GAC contactors to remove trace organic compounds via adsorption, and (5) AOP to disinfect and provide a barrier to pathogens in the water prior to recharging into the Potomac Aquifer. This AC-based AWP treatment and research facility has been in operation since 2018 and can replenish up to one million gallons per day of drinking quality water into the Potomac Aquifer. Based on the results from the research center, three full-scale SWIFT facilities are under construction, expected to all be online by 2032, increasing the daily replenishment to 100 million gallons of purified water per day (Heisig-Mitchell 2023; Water Finance & Management 2024).

The selection of an appropriate AWP framework is a critical decision that shapes the technical, economic, and operational feasibility of potable reuse projects. While both RO- and AC-based approaches have demonstrated efficacy in producing high-quality water suitable for potable reuse, they differ substantially in their treatment mechanisms, infrastructure requirements, and operational characteristics. RO-based systems excel at removing TSS and achieve very low TOC levels, making them the established standard for many potable reuse applications (Tang *et al*. 2018; Trussell & Pisarenko 2024). Conversely, AC-based systems offer advantages in energy efficiency and eliminate the need for concentrate management, though they do not remove dissolved salts (Sundaram *et al*. 2020; Tow *et al*. 2021; Peterson *et al*. 2022). The decision matrix presented in Table 1 systematically compares these two approaches across key technical, regulatory, and operational criteria to support informed decision-making for utilities evaluating AWP implementation.

### 2.2. Regulatory compliance

Regulatory frameworks for potable water reuse have evolved substantially to protect public health by addressing pathogen control through multi-barrier treatment approaches (United States Environmental Protection Agency Office of Water 2023). Of the 14 states with IPR regulations, California’s IPR regulations are considered among the most rigorous in the world, serving as a model for other states’ in-progress or implemented regulations. California’s Title 22 regulations establish the ‘12/10/10 rule’ requiring 12-log enteric virus, 10-log *Giardia*, and 10-log *Cryptosporidium* reduction for IPR, while newly effective DPR regulations mandate heightened requirements of 20/14/15 log removal value (LRV) for the same pathogens to account for the absence of environmental buffers (U.S. Environmental Protection Agency, Office of Water 2021). Within this regulatory landscape, secondary biological treatment has historically received conservative or zero pathogen removal credits. Hill *et al*. (2025)’s review examining LRVs during secondary treatment found a median LRV of 1.8 log_10_ across studied reference viruses while ranging from −0.4 to 5.3 LRV. However, when regulatory frameworks focus on 5th percentile performance to ensure reliability, these values often fall below 0.5 log_10_, explaining why conventional activated sludge and clarification systems remain generally uncredited in current DPR regulations (Hill *et al*. 2025).

While regulatory crediting is predominantly awarded to AWPFs, emerging research suggests that properly designed, operated, and monitored secondary wastewater treatment can achieve higher removal efficiencies than conventional activated sludge (Wang *et al*. 2025). This presents opportunities for additional crediting that can reduce the burden and cost of downstream AWP (Wang *et al*. 2025). Treatment intensification strategies, exemplified by Hampton Roads Sanitation District’s SWIFT facility, demonstrate how enhanced secondary and tertiary processes can serve as credited pathogen barriers within multi-barrier treatment trains (Heisig-Mitchell 2023). Similarly, membrane bioreactors (MBRs) currently receive 1.5/2/4 log removal credits for viruses, protozoa, and bacteria while offering compact footprints with 50% space reduction compared to conventional activated sludge. The integration of regulatory crediting frameworks into upstream wastewater treatment plant (WWTP) design decisions represents a point where operational optimization can both recognize existing public health protection and yield economic benefits. This approach is valuable given the regulatory divide in the U.S., where wastewater treatment (Clean Water Act; (U.S. Environmental Protection Agency Office of Water 2013)) and drinking water treatment (Safe Drinking Water Act; (U.S. Environmental Protection Agency Office of Water 2013) fall under separate regulatory jurisdictions, resulting in administrative separation between WWTPs and AWPFs. As regulatory frameworks evolve, the utilization of secondary treatment as a credited barrier within the potable reuse treatment train will increasingly influence facility planning, design, and operational decision-making.

In addition to pathogen control, regulatory frameworks are designed to protect public health from chemical contaminants. In AWPFs, most regulated chemicals (e.g., organic, inorganic, radionuclide, disinfection byproducts (DBPs), heavy metals) are not detected in treated effluent; regulated chemicals that are detected typically occur at concentrations lower than those found in conventionally treated drinking water supplies, confirmed by required ongoing monitoring and periodic sampling (National Research Council 2012). Certain water quality parameters, such as total organic carbon (TOC) and total nitrogen (TN), are regulated not only to protect public health but also to prevent interference with treatment processes. Elevated levels of these constituents can reduce treatment efficiencies by fouling membranes during AWP, reacting with disinfectants, forming DBPs, or causing eutrophication in environmental buffers. California’s IPR and DPR regulations include maximum contaminant levels (MCLs) for both TOC (≤0.5 mg/L, 20-week average or average of last four results) and TN (≤10 mg/L), while DPR regulations also include MCLs for nitrate and nitrite, ≤10 mg/L and ≤1 mg/L, respectively (Cal. Code Regs. Tit. 22 § 64431 2008; Cal. Code Regs. Tit. 22 § 64444 2008) Although regulations for these parameters are location specific and depend on the requirements of the reuse type, it is advantageous for WWTPs to implement technologies capable of nutrient removal beyond local regulations to assist AWP downstream.

### 2.3. Influence of wastewater treatment on AWP processes

The strategic upgrade of secondary wastewater processes offers substantial advantages for AWP processes, primarily through enhanced constituent removal and optimized effluent quality. Secondary treatment technologies demonstrate significant potential to remove organic and inorganic contaminants that would otherwise compromise downstream AWP, for both IPR and DPR. Facilities that utilize AC-based treatment face distinct challenges with ammonia removal. Ammonium ions, being small and strongly hydrated, exhibit weak affinity for the graphitic surface of pristine AC due to the absence of hydrophobic interactions (Halim *et al*. 2010; Karri *et al*. 2018). While AC surface modification techniques, such as acid treatment, functionalization, or surfactant loading (Vassileva *et al*. 2009; Lee *et al*. 2018), can significantly enhance adsorption capacity for ammonium ions, these techniques are rarely applied in AWPFs due to high cost and compromised regeneration potential. However, the removal of ammonia prior to disinfection is essential, due to its reactivity with UV/chlorine in which DBP formation is enhanced (Zhang *et al*. 2022). Therefore, AC-based AWP for both IPR and DPR requires nitrification in upstream secondary wastewater treatment to prevent ammonia from reaching the disinfection stage.

Importantly, even RO-based AWP systems, which effectively remove ammonia and related constituents, must also receive nitrified effluent in California DPR applications. While removal efficiency depends on conditions such as pH and constituent loading, RO can achieve 90–99% ammonia removal (Li & Jin 2022) and 85–95% nitrate removal (Duong *et al*. 2021; Popova *et al*. 2023), making it a dependable physical separation barrier prior to disinfection. However, this creates the risk that should any malfunction occur within the RO system, and ammonia is not sufficiently removed prior to disinfection, DBPs could be released into the effluent stream. To protect public health from this risk, California IPR employs the barrier of aquifer retention time (minimum 2 months by added tracer; minimum 3 months by intrinsic tracer) and dilution to manage any DBPs of concern. DPR, however, does not utilize an environmental buffer and therefore even RO-based AWP systems must receive nitrified effluent in California DPR applications. This mandated redundancy reduces membrane fouling, minimizes DPBs, and provides an additional public health barrier in the absence of an environmental buffer (Dahdah *et al*. 2025).

Relying on RO for removal of nitrate creates significant waste management challenges due to accumulation in the concentrate. Although existing facilities employ dilution strategies to meet discharge compliance, this does not reduce the total mass loading of nitrogen within municipal wastewater reuse concentrate (MWRC), which must then undergo additional treatment prior to disposal. Relying solely on AWP for nitrogen management highlights critical limitations. Implementing nitrification-denitrification (NDN) processes within upgraded secondary treatment enables WWTPs to achieve biological nitrogen removal and reduces burdens on AWPFs. Integrated NDN processes, whether single-stage or multi-stage configurations, convert nitrogen compounds to nitrogen gas, which is released into the atmosphere. This approach not only ensures compliance with potable reuse TN regulations but also minimizes concentrate management challenges and improves overall system efficiency.

Upgraded secondary treatment additionally reduces constituent loading on downstream processes by decreasing TOC concentrations – a critical consideration given that MWRC can contain TOC levels 5–6-fold higher than feed water concentrations (Finnerty *et al*. 2024). While integrating ozonation and BAC upstream of RO can facilitate partial TOC transformation and removal, these processes do not completely resolve concentrate management issues. Most importantly, high-quality secondary effluent is essential for optimal RO performance and overall system reliability. Poor effluent quality accelerates membrane fouling, resulting in increased energy consumption, elevated chemical usage, permanent loss of treatment capacity, and substantially higher long-term operation and maintenance costs (Ahmed *et al*. 2023; Tayeh *et al*. 2025). Additionally, degraded effluent quality introduces operational challenges for advanced oxidation and adsorption processes; elevated turbidity, dissolved organic carbon, ammonia, nitrite, and residual chlorine increase ozone demand and necessitate more frequent biofilter backwashing and GAC replacement cycles (Ho *et al*. 2011; Steinle-Darling *et al*. 2023). From an environmental health perspective, addressing micropollutants (including pathogenic microorganisms, metals, per- and polyfluoroalkyl substances (PFAS), DBP precursors, and others) at their highest concentrations in secondary treatment is preferable to relying solely on downstream AWP removal. While RO effectively removes many constituents, treating the compositionally variable MWRC presents significant operational challenges that can be mitigated through upstream treatment optimization.

AWPF design for potable reuse is contingent upon multiple interdependent parameters: influent source water quality characteristics, effluent quality specifications, the implementation approach (IPR vs DPR), regulatory mandates for pathogen log reduction and chemical contaminant attenuation, geographical constraints and needs (e.g., groundwater replenishment, seawater intrusion prevention), and overall cost (California State Water Resources Control Board 2019). These determinants govern the selection and configuration of individual unit processes while establishing requisite redundancy to ensure system reliability and protect public health. Notably, the quality of influent entering the AWPF is determined by upstream wastewater treatment processes, directly impacting both which AWP processes are required for potable reuse and their operational parameters (e.g., chemical dosages, membrane lifespan, treatment efficiency) (Steinle-Darling *et al*. 2023). Therefore, optimizing secondary wastewater treatment is not merely complementary to AWP but rather a strategic prerequisite for achieving cost-effective, reliable, and regulation compliant potable reuse. Failure to consider the interdependence between upstream biological treatment and downstream AWP can result in suboptimal system design, increased operational costs, and compromised treatment reliability, undermining the long-term viability and sustainability of potable reuse projects.

## 3. ENHANCEMENT OF SECONDARY TREATMENT VIA NEXT-GENERATION BIOTECHNOLOGIES

### 3.1. Limitations of conventional secondary wastewater technologies during potable reuse

**Figure 2 |:**
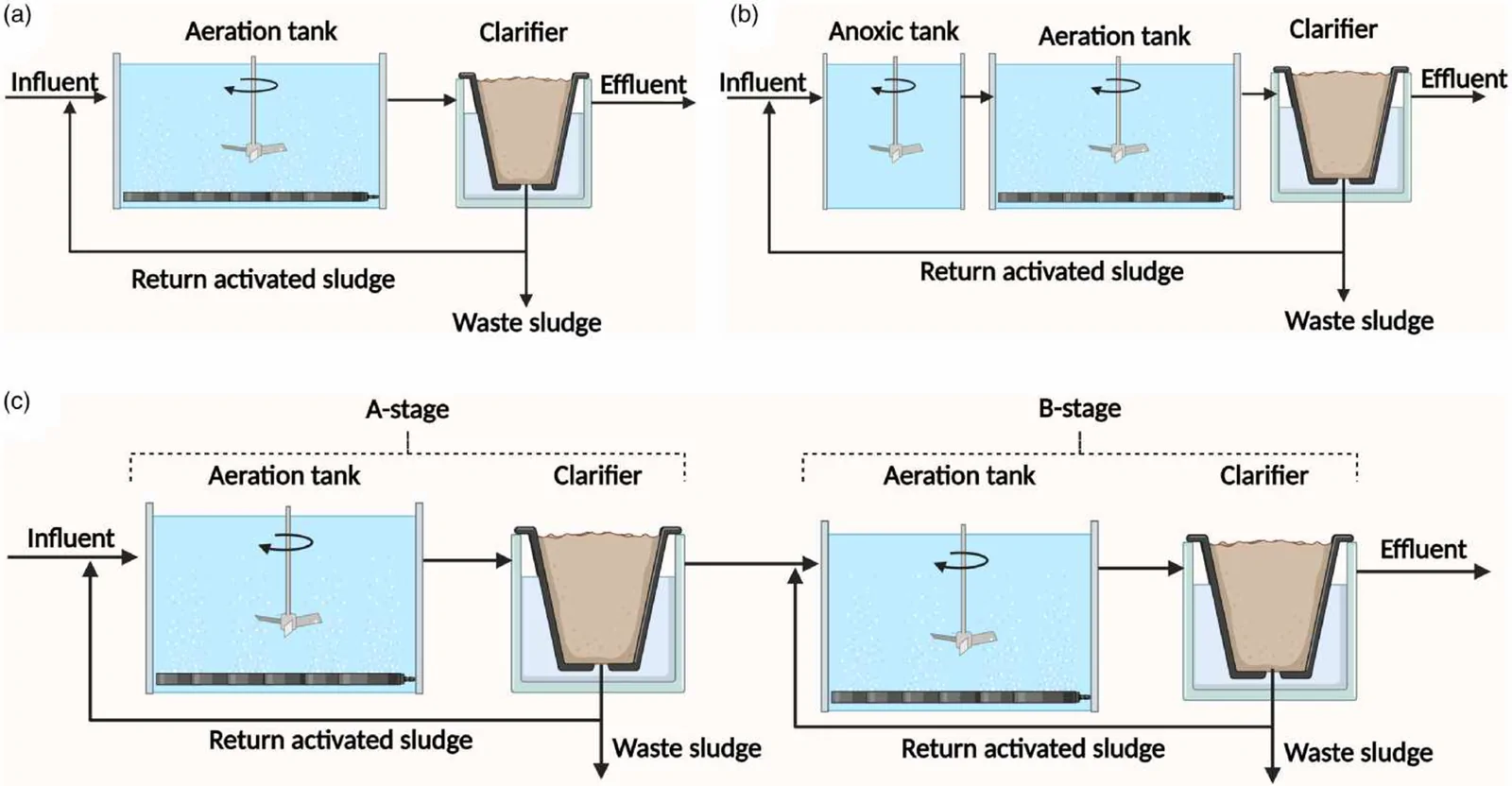
Schematic representation of conventional and advanced secondary wastewater treatment processes: (a) conventional activated sludge (CAS), (b) Modified Ludzack-Ettinger (MLE), and (c) adsorption/bio-oxidation (AB) process.

Traditional secondary wastewater treatment processes, such as conventional activated sludge (CAS) (Figure 2(a)), utilize suspended microbial growth to remove carbon, achieving over 90% TOC reduction (Kayser 2005; Silva 2023; Shamshad & Ur Rehman 2025), and can transform nitrogen via nitrification provided solids retention time (SRT) is sufficiently long. The Modified Ludzack-Ettinger (MLE) process is a more complex activated sludge process and well-established biological nutrient removal (BNR) configuration designed to achieve NDN (Figure 2(b)). This system consists of an anoxic zone for denitrification followed by an aerobic zone for nitrification, with internal recycling from the aerobic to anoxic zone (Barnard 1973). By removing nitrogen, MLE is inherently an improvement relative to CAS in potable reuse schemes. However, both CAS and MLE rely on secondary clarification for solid separation, making it susceptible to sludge bulking, leading to poor sludge settleability and biomass retention (Martins *et al*. 2004; Chen *et al*. 2015). These issues can elevate turbidity (Li & Stenstrom 2018) and allow breakthrough of pathogens (Campos *et al*. 2016; García-Fontana *et al*. 2020; Ahn *et al*. 2025) and micropollutants (Guo *et al*. 2010; Yoo & Lee 2021). Consequently, effluents from CAS and MLE systems contain residual suspended solids, organic matter, and microbial contaminants that foul downstream microfiltration membranes, increasing chemical cleaning demands, and shortening membrane lifespan.

The adsorption/bio-oxidation (AB) process is a two-stage variation of the activated sludge process that combines rapid adsorption with slower biological oxidation, allowing for high organic loading yet a smaller footprint than CAS (Figure 2(c)). The A-stage features a high-rate activated sludge (HRAS) process that is designed for organics removal, followed by a low-loaded B-stage that focuses on nutrient removal (Böhnke 1995). By operating at short hydraulic retention time (HRT) and SRT, the A-stage maximizes waste activated sludge generation, and consequently energy recovery opportunities in anaerobic digestion (Modin *et al*. 2016). Concurrently, this drives a reduction in required oxygen by limiting oxidation of organics, reducing the most significant energy consuming process in WWTPs (Gude 2015; Drewnowski *et al*. 2019). Furthermore, biogas generated from HRAS has a greater methane yield than waste activated sludge from CAS (Trzcinski *et al*. 2016; Rahman *et al*. 2019), and primary sludge (Abdelrahman *et al*. 2022). The main appeal of the AB process lies in this energy recovery advantage (Liu *et al*. 2019b). However, a considerable limitation of the AB process is the carbon removal in the A-stage, which results in limited electron donors for denitrification in the B-stage.

While processes such as CAS, MLE, and AB systems achieve substantial reductions in carbon and can provide nitrogen transformation or removal, their effluents often contain residual suspended solids, dissolved organics, nutrients, and microbial contaminants that challenge downstream AWP processes. These limitations, coupled with high aeration energy demands, excess sludge production, and instability under variable loading, highlight the need for further improvement. Consequently, modern WWTPs are adopting next-generation secondary treatment approaches that integrate advanced materials, novel microbial metabolisms, and innovative reactor designs to enhance treatment efficiency and water quality, reduce energy demands and physical footprint, and enable energy and resource recovery.

### 3.2. Emerging next-generation biotechnologies to enhance secondary wastewater treatment

**Figure 3 |:**
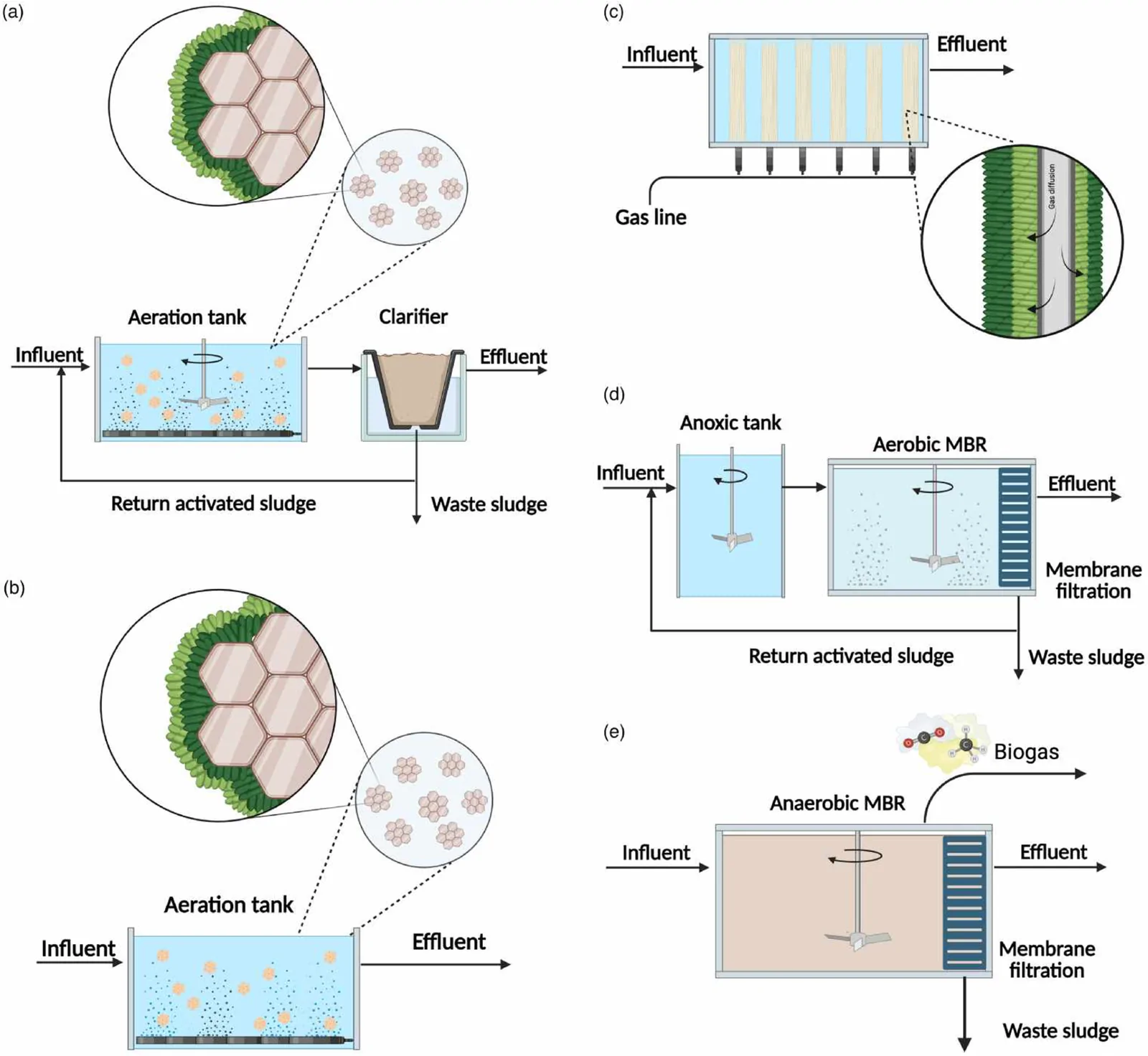
Schematic representation of next-generation biotechnologies, (a) integrated fixed-film activated sludge (IFAS) (b) moving bed bioreactor (MBBR), (c) membrane biofilm reactor (MBfR), (d) Modified Ludzack-Ettinger membrane bioreactor (MLE-MBR), (e) anaerobic membrane bioreactor (AnMBR).

Emerging biotechnologies, specifically integrated fixed-film activated sludge (IFAS) and the moving bed biofilm reactor (MBBR), membrane biofilm reactor (MBfR), hybrid Modified Ludzack-Ettinger membrane bioreactor (MLE-MBR), and anaerobic membrane bioreactor (AnMBR) (Figure 3(a)–3(e)), have demonstrated enhanced secondary treatment performance while meeting wastewater effluent regulations for environmental discharge. However, their compatibility with AWP frameworks remains insufficiently evaluated. Therefore, continued evaluation is needed to determine how these systems integrate with downstream AWP and whether they can reliably achieve compliant effluent quality.

#### 3.2.1. Attached-growth biotechnologies

Biofilm technologies utilize fixed or moving biofilm carriers to support microbial colonization for carbon and nitrogen removal during secondary wastewater treatment. Unlike suspended-growth systems, attached-growth systems retain biomass on media, which increases volumetric treatment capacity, provides operational flexibility, and reduces required HRT (Saini *et al*. 2023). Further, the protective biofilm matrix, particularly the extracellular polymeric substances (EPS), enhances resistance to toxic shock loads and operational fluctuations by shielding microorganisms from environmental perturbations and providing a stable microenvironment (Al-Amshawee *et al*. 2021). The prolonged SRT due to attached growth results in reduced sludge production relative to CAS. Notably, these biotechnologies establish a unique microenvironment wherein simultaneous aerobic and anoxic conditions can occur within a single reactor, attributable to three-dimensional concentration gradients formed between the attached media and bulk liquid phase. This spatial stratification can enable simultaneous nitrification and denitrification (SND) with reduced footprint relative to processes such as MLE (Li & Zhang 2018; Werkneh 2022). Emerging biofilm biotechnologies such as IFAS, MBBRs, and MBfRs have been reported to achieve high carbon and nitrogen removal (Waqas *et al*. 2020; Chang *et al*. 2022; Ravishankar *et al*. 2022; Zhao *et al*. 2023; Zhong *et al*. 2024).

IFAS systems retrofit CAS with suspended biofilm carriers, combining the advantages of suspended- and attached-growth processes. The coexistence of attached and suspended biomass increases microbial diversity, while providing resilience even at low temperatures (#15 °C) and under variable nutrient loads (Kim *et al*. 2011). MBBRs similarly employ suspended biofilm carriers, but rely exclusively on attached biomass, with no intentional suspended biomass (i.e., no return activated sludge). As a purely attached-growth process, the treatment performance of the MBBR depends on the amount of biofilm surface area, carrier fill fraction, and biofilm activity, thereby allowing for a smaller footprint. Both IFAS and MBBR systems can be configured as single- or multi-stage systems to enhance nitrogen removal consistency in advanced applications, such as the MLE or AB processes (Gu *et al*. 2017; Xu *et al*. 2017; Liu *et al*. 2019b). However, both technologies face CAS-related challenges such as mechanical mixing or high aeration requirements to retain free-floating media and foaming (Kim *et al*. 2011; Rosso *et al*. 2011; Gellner 2019).

The MBfR is an advanced biotechnology that uses porous membranes to supply gaseous substrates into a biofilm formed on the membrane’s exterior surface, reducing energy demands for aeration. This counter-diffusional process accomplishes contaminant removal by supplying the electron donor and acceptor from opposite sides of the biofilm: gaseous substrate through the membrane lumen and dissolved substrate from the bulk liquid. Unlike membrane filtration processes, the hollow-fiber membranes within MBfRs are not designed for physical separation and colloid removal but instead provide a highly controlled gas diffusion technique that also provides increased surface area to encourage biofilm formation. MBfRs offer high gas utilization efficiency, low energy consumption, and small reactor footprints (Martin & Nerenberg 2012). Oxygen-based MBfRs, or membrane aerated biofilm reactors (MABRs), deliver oxygen through hollow fiber membranes without forming bubbles, preventing surface foaming, shear forces, and volatile organic carbon stripping while achieving oxygen transfer efficiencies up to 100% (Kavousi *et al*. 2016; Li & Zhang 2018). This bubble-free operation provides significant cost and energy savings (40–70%) relative to CAS (Martin & Nerenberg 2012). Ideal for SND, MABRs have been reported to achieve 60–92% TN and 92–96% COD removal, respectively (Ravishankar *et al*. 2022; Zhao *et al*. 2023; Zhong *et al*. 2024). Importantly, to ensure high removal efficiency, oxygen delivery and C/N ratio must be considered and controlled properly. Higher C/N ratios provide sufficient electron donors for denitrification without inhibiting nitrification. However, temporal variability in influent strength can necessitate adjustment of oxygen to prevent an oversupply, which inhibits denitrification (Li & Zhang 2018; Ravishankar *et al*. 2022; Ghasemi *et al*. 2024). MBfRs can also be operated using hydrogen or methane as gaseous electron donors, which has been explored at the bench scale, however MABRs have been more prominently seen at the pilot-scale for nitrogen removal (Kim *et al*. 2011; Wei *et al*. 2012; Shreve & Brennan 2019; Chang *et al*. 2022; Dicataldo *et al*. 2025).

#### 3.2.2. Combining biological treatment with membrane separation

Membrane bioreactors (MBRs) incorporate microfiltration or ultrafiltration membranes that act as a physical barrier for biomass retention and the separation of suspended solids, bacteria, and pathogens. Integration of MBR with MLE (i.e., anoxic zone followed by an aerobic membrane bioreactor) has been adopted in potable reuse treatment trains because it integrates efficient BNR with advanced solid-liquid separation. This hybrid configuration (MLE-MBR) bypasses the secondary clarification step and can be directly coupled with downstream RO. Moreover, MBRs operate at longer solid retention times (SRTs) and at higher mixed liquor suspended solids (MLSS) concentrations, promoting the growth and retention of diverse microbial consortia including slow-growing microorganisms. This feature can enhance degradation of slowly biodegradable or recalcitrant compounds, enhancing BNR efficiency and stability under varying loading conditions (Côté *et al*. 1997; Rosenberger *et al*. 2002; Holland *et al*. 2006; Tam *et al*. 2006; Barreto *et al*. 2017). MLE-MBRs can remove more than 99% TSS, producing effluent with turbidity typically below 0.3 nephelometric turbidity units (NTUs; (Côté *et al*. 1997; Holland *et al*. 2006). Additionally, 4 to 7 log removal values (LRVs) of fecal coliforms, P. aeruginosa, and bacteriophages can be achieved (Côté *et al*. 1997; Holland *et al*. 2006; Ng *et al*. 2019). From an operational perspective, the compact footprint and elimination of secondary clarifiers make the MLE-MBR configuration particularly attractive for utilities facing space limitations or retrofitting constraints.

Anaerobic membrane bioreactors (AnMBRs) similarly couple membrane filtration with biological treatment but operate under fully anaerobic conditions. This system enables energy recovery through methane production, reduces energy demand by eliminating aeration, and significantly decreases sludge production due to the lower biomass yields of anaerobic microorganisms (Smith *et al*. 2012; Song *et al*. 2018; Robles *et al*. 2021; Mahmood *et al*. 2022). As emerging mainstream biotechnologies, AnMBRs achieve high carbon removal efficiencies (COD removal >85%) with low sludge yields (0.19–0.26 g MLSS g^−1^ COD removed at 25 °C) (Smith *et al*. 2012; Robles *et al*. 2021, 2022). As with MLE-MBR, the use of microfiltration or ultrafiltration membranes results in nearly complete TSS removal, potentially providing compatibility with downstream RO in certain scenarios (i.e., Orange County Ground Water Replenishment System, Orange County, CA). However, dissolved methane in anaerobic effluents represents a lost energy source and potent greenhouse gas if not recovered (Crone *et al*. 2016). Although progress has been made on energy efficient recovery processes, biological oxidation (Hatamoto *et al*. 2010), and even utilizing methane during nitrogen removal (Liu *et al*. 2020), dissolved methane remains a significant barrier to AnMBR adoption.

### 3.3. Implementation considerations for next-generation biotechnologies for potable reuse

#### 3.3.1. Nutrient removal

Regulated nutrient removal in potable reuse systems is determined by the level of associated human health risk, thereby nitrogen removal is a primary regulatory focus due to high human health risks from DBPs such as NDMA, while phosphorus removal is not emphasized given its low human health risk. To be implementable within a potable reuse treatment train, next-generation biotechnologies must meet nutrient removal requirements prior to AWP. Currently, California IPR utilizing RO-based AWP is the only potable reuse treatment train that does not require upstream nitrification; all other configurations must maintain nitrification capacity within secondary treatment to ensure AWP efficiency and public health safety barriers. Consequently, most WRRFs implement upgraded secondary biotechnologies that incorporate nitrogen removal processes (i.e., NDN, SND) to achieve biological nitrogen removal (Zahreddine & Nepal 2021). This approach not only meets potable ruse TN regulations but also improves overall AWP system efficiency by reducing contaminant loading on activated carbon or RO process, allowing these units to target non-biodegradable constituents more effectively. Although not required for RO-based IPR, implementing nitrification and NDN capabilities provides WWTPs with enhanced downstream efficiency and operational flexibility to adapt to evolving regulations should future nitrification ever be required.

Conventional nitrification proceeds through two sequential reactions in an aerobic environment, where autotrophic bacteria oxidize ammonia into nitrite, followed by further oxidation to nitrate. However, to achieve high rates of ammonia oxidation in suspended-growth systems such as CAS, high aeration and mixing rates are required to ensure adequate dissolved oxygen levels and uniform distribution throughout the reactor volume (Daigger 2014). Implementing attached-growth processes circumvents this issue, with increased ammonia oxidizing and nitrite oxidizing bacterial activity (Kim *et al*. 2011; Rosso *et al*. 2011), however suspended carriers such as those used in IFAS and MBBRs can undesirably lead to increased aeration/energy demands to ensure biofilm carrier suspension (Rosso *et al*. 2011). Due to the fixed nature of the membrane modules, MABRs eliminate the need for aeration recirculation or mechanical mixing while maximizing oxygen transfer efficiencies (OTEs) through direct gas-to-biofilm diffusion, achieving OTEs exceeding 80%, thereby reducing overall energy requirements to approximately one-fifth that of CAS (Campo *et al*. 2024), while maintaining nitrification efficiencies above 93% (Corsino & Torregrossa 2022).

NDN requires both aerobic and anoxic biochemical environments for the occurrence of nitrification and denitrification, respectively, accomplished by systems such as the MLE-MBR or the MBBR when operated as a two-stage aerobic/anoxic system providing effective carbon and nitrogen removal (Odegaard 2006; Madan *et al*. 2022). Reducing footprint to a single reactor, IFAS, MBBR (single-stage), and MABR systems have been reported to complete SND and can support novel shortcut nitrogen removal metabolisms, such as anammox (Zhao *et al*. 2022; Vilardi *et al*. 2023; Cui *et al*. 2025), with MABRs achieving upwards of 81% TN removal while only producing 0.06% nitrous oxide, a common SND byproduct (Corsino & Torregrossa 2022; Cui *et al*. 2025). While suspended growth systems can also support SND at HRTs as low as 4hr (Shao *et al*. 2024), removal performance typically underperforms compared to attached-growth processes. Importantly, of the next-generation biotechnologies, AnMBRs provide negligible nutrient removal, limiting compatibility with AWP requiring nitrified effluents as source water. AnMBR could theoretically be coupled with a downstream biological process for nitrogen removal, however this inevitably increases overall system complexity. Therefore, implementing an AnMBR (without additional downstream processes) into a potable reuse treatment train would only be feasible for RO-based IPR.

#### 3.3.2. Biotechnology implementation in new and existing systems

**Figure 4 |:**
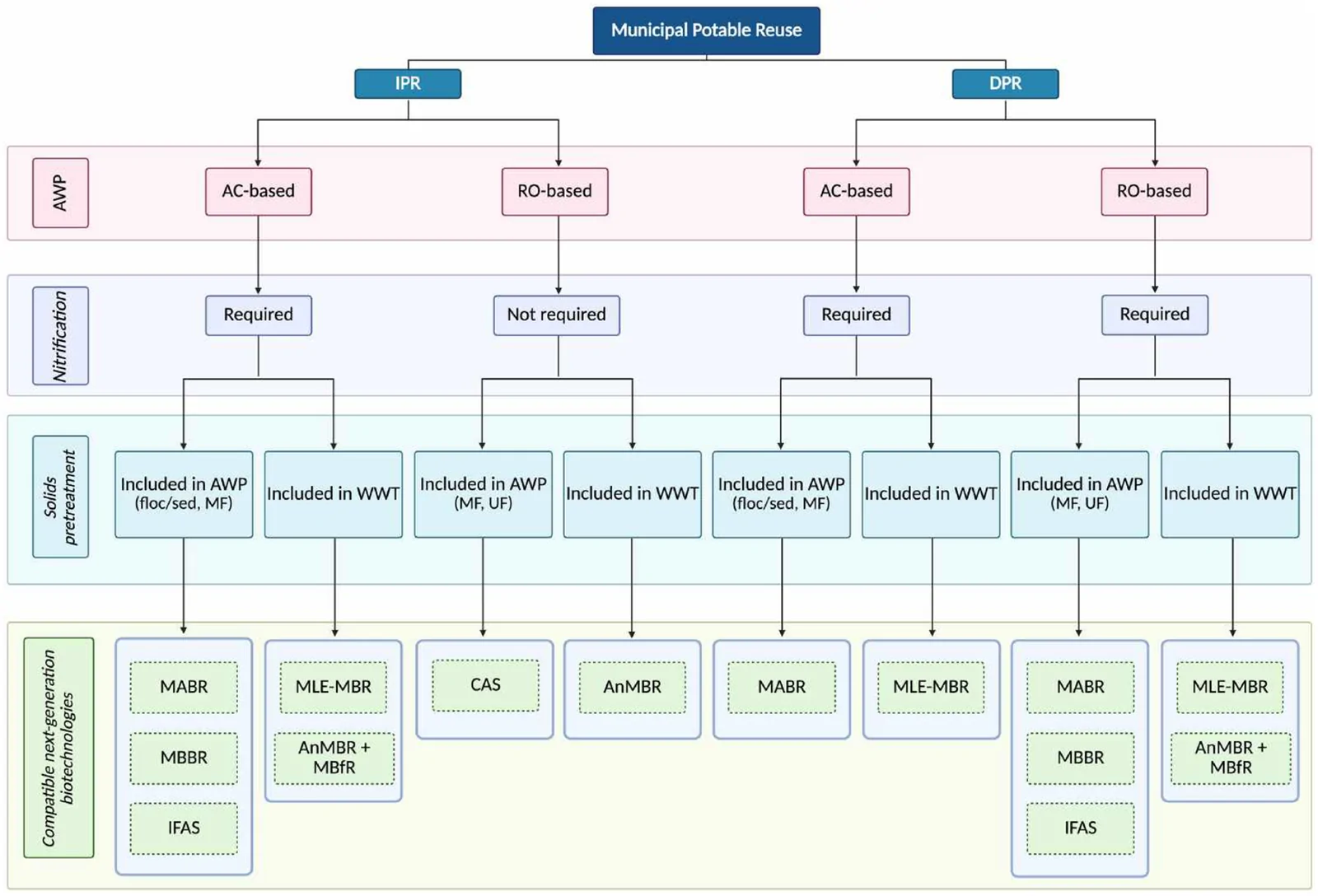
Factors influencing next-generation biotechnology compatibility within potable reuse treatment trains.

When evaluating next-generation biological treatment technologies, it is essential to consider how each is integrated into a secondary wastewater treatment train. IFAS systems, which combine attached and suspended biomass, can be installed in virtually any aeration basin that is followed by a secondary clarifier to maintain a return activated sludge (RAS) stream. MBBRs offer greater placement flexibility: they can be incorporated into either aerobic or anoxic zones, depending on the targeted biofilm function, making them suitable for retrofitting MLE or AB configurations that require additional surface area for microbial activity to increase capacity. While MABRs are typically deployed as standalone systems, they can also be adapted to existing basins if the diffused aeration system is retrofitted so that airflow is directed into the membrane modules (Shechter *et al*. 2020). It is important to note that, unlike IFAS, both MBBR and MABR configurations operate without RAS and therefore do not rely on secondary clarification for biomass retention. However, a downstream solids-separation process (e.g., floc/sed, MF, UF) is still required to remove residual suspended solids and any biofilm that may slough from the reactor media. Conversely, the use of MF or UF membranes in MLE-MBRs and AnMBRs results in nearly complete TSS removal, potentially providing compatibility with downstream RO AWP. Therefore, while attached-growth biotechnologies offer enhanced nutrient removal within the footprint constraints of retrofitted systems, their compatibility relies heavily on solids pretreatment at the AWPF, while MLE-MBRs and AnMBRs bypass those additional steps and can instead be directly coupled with downstream processes, as shown in Figure 4.

However, solids separation is only one of many criteria utilities must consider when retrofitting preexisting or establishing new systems. Table 2 systematically compares these five approaches across key technical and operational criteria to support informed decision-making when evaluating next-generation biotechnologies. Importantly, due to the emerging status of MBfRs, MLE-MBRs, and AnMBRS, specific long-term energy requirements remain poorly understood. Future research is therefore critical to quantify energy demands and develop strategies to reduce carbon footprint.

### 3.4. Opportunities to advance next-generation biotechnologies for potable reuse

Secondary treatment processes in potable reuse schemes must contribute to carbon and nitrogen removal to provide regulatory compliance. However, recent engineering advances and implementation of novel microbial metabolisms have expanded the scope of available biotechnologies. Understanding these next-generation technologies’ metabolic capabilities enables facilities to optimize treatment performance while minimizing energy use and operational costs.

#### 3.4.1. Novel metabolisms for nutrient removal

Over the past decade, several sustainable nitrogen-removal technologies have emerged, yet due to performance inconsistencies, their adoption has been gradual despite potential to reduce energy demand, chemical consumption, solids production, and overall costs. Among these innovations, partial nitritation-anammox (PNA; also referred to as deammonification), an NDN shortcut, linking partial nitritation with anaerobic ammonia oxidation (anammox), has been the most transformative, leveraging novel microbial metabolisms to achieve nitrogen removal with substantially lower oxygen and carbon requirements (Zahreddine & Nepal 2021). By converting roughly half of influent ammonia to nitrite and allowing anammox bacteria to oxidize the remaining ammonia under anoxic conditions, PNA can reduce aeration energy by 60% or more and eliminate supplemental carbon addition up to 100%. However, PNA can be challenging to achieve due to the low dissolved oxygen concentrations required to ensure NOB out competition, requiring finely controlled aeration. MABRs therefore hold potential for PNA by offering a controlled aeration environment (Wagner *et al*. 2018; Wagner *et al*. 2022; Li *et al*. 2023) and reducing overall system oxygen consumption by 25–60% compared to conventional processes. It is important to note that MABRs have been found to be dependent on pH, influent ammonia loading, temperature, and HRT, creating inconsistent PNA performance (Vesuwe *et al*. 2025). Likewise, IFAS and MBBR systems also face difficulties in consistently suppressing NOB while retaining slow-growing anammox populations under variable conditions, which has constrained full-scale implementation despite continued research efforts and pilot-scale demonstrations (Zahreddine & Nepal 2021).

Partial denitrification-anammox (PdNA; also referred to as partial nitrification-anammox-partial denitrification or PN/A/PD) represents another promising novel nitrogen removal pathway that has been at the forefront of optimizing attached-growth technologies. Achieving substantial operational aeration and carbon savings (50 and 80% reduction, respectively) compared to CAS processes (Bachmann *et al*. 2025), PdNA systems achieve high total nitrogen removal without requiring NOB suppression. Despite this benefit, only a few confirmed full-scale mainstream PdNA applications are currently operational, including installations in Virginia, USA (Bachmann *et al*. 2025). However, pilot-scale studies have demonstrated the technology’s potential, with MBBRs and MBfRs achieving PdNA efficiencies exceeding 88% (Li *et al*. 2022a; Klaus *et al*. 2023), while IFAS systems have shown lower efficiencies of approximately 38% (Ladipo-Obasa *et al*. 2022). Nevertheless, IFAS configurations remain attractive for full-scale anammox implementation due to their ability to retrofit existing activated sludge systems at minimal cost.

Uniquely, MBfRs offer the ability to couple nutrient removal with alternative gaseous electron donors/acceptors. For example, ammonia removal can be coupled with methane oxidation via nitrite/nitrate-dependent anaerobic methane oxidation (n-DAMO), achieving upwards of 85% methane and 99% nitrogen removal (Liu *et al*. 2019a; Liu *et al*. 2020). Further, for waste streams with high TOC, the MABR can be coupled with a mainstream anaerobic biotechnology, such as the AnMBR, for carbon and nitrogen removal via the Sulfate reduction Autotrophic denitrification Nitrification Integrated (SANI^®^) process. This two-stage system shows resistance to influent variability, as this process has been reported to remove over 86–95% COD and >92% TN while reducing energy consumption and sludge production (30%, 60–70%, respectively) compared to CAS (Wu *et al*. 2016; Han *et al*. 2023; Han *et al*. 2025).

#### 3.4.2. Fate of micropollutants

The AWP decision matrix demonstrates that RO-based systems excel at removing TSS, trace organic contaminants, and micropollutants, producing product water that meets potable reuse standards; however, this performance simultaneously generates the critical challenge of MWRC management. MWRC composition represents secondary effluent that has been processed through UF and/or MF, amended with low concentrations of process chemicals (0.5–2 mg/L coagulants or anti-scalants), and subsequently concentrated 5–6 fold through RO filtration (Finnerty *et al*. 2024). RO rejects a broad spectrum of dissolved and colloidal constituents, producing MWRC that typically represents 15–20% of the influent flow and contains elevated concentrations or TSS (3,000–8,000 mg/L), major ions (Na^+^, Cl^−^, Ca^2+^, ${\text{SO}}_{4}^{2-}$), nutrients (${\text{NH}}_{4}^{+}$, ${\text{NO}}_{3}^{-}$, ${\text{PO}}_{4}^{3-}$), metals, and residual process chemicals (Finnerty *et al*. 2024). MWRC also contains refractory and emerging micropollutants, including pharmaceuticals and personal care products (PPCPs), endocrine-disrupting compounds (EDCs), PFAS, and micro-plastics, that can exceed aquatic toxicity thresholds (Cemre Birben *et al*. 2017; Valdés *et al*. 2021; Finnerty *et al*. 2024). Moreover, RO performance itself is vulnerable to efficiency loss as a result of organic fouling, colloidal fouling, biofouling, and mineral scaling (Ahmed *et al*. 2023), creating a bidirectional dependency between upstream treatment quality and downstream concentrate management. Understanding MWRC composition is essential for utilities to develop integrated approaches that optimize both product water quality and concentrate management strategies, particularly for inland communities where ocean disposal is not feasible and the advantages of AC-based systems that eliminate concentrated waste streams become increasingly compelling. However, even AC-based AWPFs must manage these contaminants and consider residual disposal accordingly.

Micropollutant removal remains a critical challenge in wastewater treatment, as trace organic contaminants (TrOCs) and other chemicals of emerging concern (CECs), including pharmaceuticals, PFAS, metallic trace elements and PAHs, persist through conventional systems and accumulate in waste streams, posing ecological and public health risks. Recent literature demonstrates that next-generation biotechnological processes may offer enhanced attenuation of these compounds. IFAS systems have shown potential for improved TrOC removal (Shreve & Brennan 2019), while MBRs routinely achieve high pharmaceutical removal efficiencies exceeding 80–99% (Adeoye *et al*. 2024). Anaerobic processes, such as AnMBRs, have also gained attention for degrading compounds typically resistant to aerobic treatment, such as sulfamethoxazole, trimethoprim, clozapine, triclocarban, and amitriptyline, with further gains possible through sorptive media or multi-stage configurations (Harb *et al*. 2019). Additional mechanisms, such as ammonia-driven co-metabolic oxidation in MABRs may bolster micropollutant transformation in addition to biofilm sorption (Li *et al*. 2022b). Given the persistence, toxicity, and bioaccumulation potential of many micropollutants, their variable partitioning into biosolids depending on physiochemical and operational conditions (Venegas *et al*. 2021), a multi-barrier approach remains essential. Given these concerns, fate biodegradation potential of micropollutants across next-generation secondary treatment processes is an important consideration. Further research is therefore critical to elucidate how next-generation biotechnologies can mitigate micropollutants within potable reuse systems.

## 4. CONCLUSION

This perspective highlights that as secondary wastewater treatment has advanced to include next-generation biotechnologies that, collectively, represent a spectrum of approaches that balance treatment performance, operational complexity, energy demands, and infrastructure requirements, with no single solution universally optimal across all potable reuse contexts. Each next-generation biotechnology offers unique strengths and trade-offs in potable reuse applications. MLE-MBRs provide unmatched effluent quality and nutrient removal but are energy-intensive due to aeration and membrane fouling control. AnMBRs offer energy recovery and low sludge generation, however, they likely require extensive post-treatment for compatibility with downstream AWP. IFAS systems enhance preestablished CAS treatment resilience and nutrient removal through hybrid suspended and attached growth at minimal installation costs, while MBBRs deliver compact, stable performance with strong organic and nitrogen biodegradation, though full nutrient removal often requires integration with additional processes and requires secondary clarification or downstream membrane filtration for biomass separation. MABRs stand out for their high oxygen transfer efficiency and SND at reduced energy costs, yet remain limited by scalability and cost barriers. Next-generation biotechnology selection for potable reuse treatment trains is largely governed by nutrient removal requirements, implementation ease, and flexibility to exceed regulations for enhanced downstream AWP performance. From these criteria, the MLE-MBR is currently the most promising technology for potable reuse systems though robust nitrogen removal and suspended solids removal. However, each of these biotechnologies hold long-term potential for energy-neutral, resource-recovery-based treatment trains that can be strategically implemented to be compatible with downstream AWP. As climate pressures intensify and potable reuse evolves, the development of hybrid configurations and adaptive metabolic processes will be essential to optimize both secondary treatment performance and downstream AWP compatibility while advancing toward sustainable, circular water systems.

## Figures and Tables

**Table 1 |:** AWP decision matrix (Tang *et al*. 2018; Sundaram *et al*. 2020; Guarin & Pagilla 2021; Scholes *et al*. 2021; Tow *et al*. 2021; Peterson *et al*. 2022; Walker *et al*. 2023)

Criteria	RO-based	AC-based
Common Configuration	MF-RO-UV/AOP-Stabilization-Disinfection	Floc/sed-O.-BAF-GAC-UV-Disinfection
	**Excellent**	**Good**
Regulatory acceptance	Standard treatment in potable reuse; well accepted by regulators for IPR/DPR	Established approach with proven track record
	**Excellent**	**Poor**
TDS removal	Good removal of TDS and majority of chemical contaminants through RO process	Ozone/BAF does not remove TDS
	**Excellent**	**Moderate**
TOC	Very low TOC levels in treated water	Higher levels of TOC compared to membrane systems
	**Poor**	**Excellent**
Energy efficiency	Energy intensive due to high-pressure RO process	More energy efficient than RO-based systems
	**Poor**	**Excellent**
Brine management	Disposal of concentrated brine challenging, especially for inland communities	Eliminates need to manage highly concentrated brine reject streams
	**Excellent**	**Excellent**
Public health protection	Multiple barrier approach with proven pathogen removal	Equally protective through multi-barrier approach with biological and chemical treatment
	**Excellent**	**Good**
Scalability	Several successful full-scale implementations for IPR/DPR applications	Proven at various scales with growing adoption
	**Moderate**	**Moderate**
Maintenance requirements	Membrane replacement, fouling management, chemical cleaning cycles	GAC regeneration required; ozone system maintenance; BAF backwashing
Advantages	Well accepted by IPR/DPR regulations Good removal of TDS and most chemical contaminants	More energy efficient than RO-based No brine and associated challenges Equally protective of public health through multi-barrier approach
Disadvantages	Energy intensive Unavoidable brine production which requires further treatment and disposal (challenging for inland communities)	GAC regeneration Does not remove TDS Higher levels of TOC

**Table 2 |:** Next-generation biotechnology decision matrix (Côté *et al*. 1997; Holland *et al*. 2006; Kim *et al*. 2011; Rosso *et al*. 2011; Smith *et al*. 2012; Wei *et al*. 2012; Kavousi *et al*. 2016; Wagner *et al*. 2018; Gellner 2019; Shreve & Brennan 2019; Liu *et al*. 2020; Shechter *et al*. 2020; Robles *et al*. 2021, 2022; Zahreddine & Nepal 2021; Chang *et al*. 2022; Corsino & Torregrossa 2022; Ladipo-Obasa *et al*. 2022; Madan *et al*. 2022; Ravishankar *et al*. 2022; Wagner *et al*. 2022; Zhao *et al*. 2022, 2023; Han *et al*. 2023; Vilardi *et al*. 2023; Campo *et al*. 2024; Ghasemi *et al*. 2024; Shao *et al*. 2024; Zhong *et al*. 2024; Bachmann *et al*. 2025; Cui *et al*. 2025; Dicataldo *et al*. 2025; Han *et al*. 2025; Vesuwe *et al*. 2025)

Criteria	IFAS	MBBR	MBfR/MABR	MLE-MBR	AnMBR
Biomass Type	Attached + suspended	Attached only	Attached only	Suspended	Suspended
Technology Maturity	Established	Established	Emerging	Emerging	Emerging
*Infrastructure and Management*					
	**Excellent**	**Good**	**Moderate**	**Moderate**	**Moderate**
Implementation Flexibility	Retrofits an aeration basin	Can be added to aerobic or anoxic zones in MLE/AB systems	Typically standalone; can retrofit basins if aeration redirected	Requires MLR and membrane infrastructure	Requires fully anaerobic system and membrane infrastructure
	**Poor**	**Poor**	**Excellent**	**Moderate**	**Excellent**
Energy Efficiency	High aeration and mechanical mixing requirements	High aeration demands to maintain carrier suspension	Very low energy (~⅕ CAS) due to non-bubbling aeration	Moderate aeration needs; Requires membrane fouling management	No aeration required; energy recovery through biogas production
*Treatment Performance*					
	**Excellent**	**Excellent**	**Excellent**	**Excellent**	**Poor**
Nitrogen Removal	NDN and SND capable; supports anammox; enhanced nitrifying bacterial activity	NDN (two-stage) or SND (single-stage); supports anammox; enhanced nitrifying bacterial activity	Outstanding SND in single reactor; supports anammox and enhanced nitrifying bacterial activity	Effective NDN via MLE configuration	Negligible nitrogen removal; not compatible with nitrification requirements; limits potable reuse applicability
	**Poor**	**Moderate**	**Moderate**	**Excellent**	**Excellent**
Solids Removal	Requires secondary clarifier followed by additional solids treatment (e.g., floc/sed, MF)	Bypasses secondary clarifier yet requires additional solids treatment (e.g., floc/sed, MF)	Bypasses secondary clarifier yet requires additional solids treatment (e.g., floc/sed, MF)	Nearly complete TSS removal due to membrane retention	Nearly complete TSS removal due to membrane retention
*Potable Reuse Compatibility*					
	**Good**	**Good**	**Good**	**Excellent**	**Poor**
AC AWP	Requires additional solids treatment (e.g., floc/sed, MF)	Requires additional solids treatment (e.g., floc/sed, MF)	Requires additional solids treatment (e.g., floc/sed, MF)	Direct coupling possible (bypass clarification)	Does not meet nitrification requirements
	**Good**	**Good**	**Good**	**Excellent**	**Excellent**
RO AWP: IPR	IPR and DPR nitrogen removal requirements met; Requires additional solids treatment (e.g., floc/sed, MF)	IPR and DPR nitrogen removal requirements met; Requires additional solids treatment (e.g., floc/sed, MF)	IPR and DPR nitrogen removal requirements met; Requires additional solids treatment (e.g., floc/sed, MF)	Direct coupling possible: nutrient and solids removal requirements met	Direct coupling possible for CA IPR;
					**Poor**
RO AWP: DPR					Nitrification requirements for DPR not met
	**Good**	**Good**	**Excellent**	**Moderate**	**Moderate**
Emerging Capabilities: Shortcut N Removal	Shortcut pathway capable (PdNA demonstrated full-scale); attached-growth carriers allow for biofilm co-communities; Supported metabolisms decrease N.O emissions	Shortcut pathway capable (e.g., anammox); attached-growth carriers allow for biofilm cocommunities	Shortcut pathway capable (e.g., anammox, n-DAMO); highly controlled aeration rates supports complex biofilm cocommunities; Supported metabolisms decrease N O emissions and can lower CH, emissions if coupled with AnMBR	Possible, but requires exploration using this configuration	Nitrification metabolisms not supported, but dissolved CH, can support novel metabolisms when coupled with downstream MBfR
Advantages	Can retrofit existing infrastructure Enhances nitrogen removal with increased microbial diversity Supports novel communities	Suitable for MLE/AB retrofits Flexible placement in aerobic or anoxic zones No RAS Enhanced N removal Supports novel communities	Exceptional energy efficiency Small footprint No foaming challenges or VOC stripping Excellent nitrogen removal including SND in single reactor	Integrated BNR with advanced filtration Direct RO coupling possible Nearly complete TSS removal High pathogen removal	Energy recovery via methane production No aeration required Very low sludge production Nearly complete TSS removal
Disadvantages	High energy demands for aeration and mixing Foaming challenges Requires RAS + secondary clarifier Requires downstream solids removal	High aeration required for carrier suspension Foaming challenges Requires downstream solids removal	Critical oxygen delivery and C/N ratio control Influent variability requires adjustment MABR at pilot scale (limited establishment)	Membrane fouling management required Moderate to high energy requirements for aeration and membrane cleaning Laboratory scale (limited establishment)	Incompatible with nitrogen requirements Dissolved CH_4_ decreases energy recovery and increases carbon footprint

## Data Availability

All relevant data are included in the paper or its Supplementary Information.

## HIGHLIGHTS

- Attached-growth biotechnologies offer enhanced nitrogen removal.
- Membrane bioreactors result in nearly complete suspended solids removal, offering compatibility with RO.
- Novel nitrogen metabolisms may improve the efficiency of conventional nitrification-denitrification but remain relatively untested in potable reuse systems.
- MLE-MBRs are currently the most promising biotechnology for potable reuse systems.
